# How elimination of lymphatic filariasis as a public health problem in the Kingdom of Cambodia was achieved

**DOI:** 10.1186/s40249-018-0394-7

**Published:** 2018-02-20

**Authors:** Virak Khieu, Vandine Or, Chhakda Tep, Peter Odermatt, Reiko Tsuyuoka, Meng Chuor Char, Molly A. Brady, Joshua Sidwell, Aya Yajima, Rekol Huy, Kapa D. Ramaiah, Sinuon Muth

**Affiliations:** 1 National Centre for Parasitology, Entomology and Malaria Control, Ministry of Health, 477 Betong Street (Corner St.92), Village Trapangsvay, Sanakat Phnom Penh Thmey, Khan Sensok, Phnom Penh, Cambodia; 2 Directorate General for Health, Ministry of Health, 80, Samdech Penn Nouth Blvd. (289), Sangkat Boeungkak 2, Tuol Kork District, Phnom Penh, Cambodia; 3 Department of Epidemiology and Public Health, Swiss Tropical and Public Health Institute, Basel, Switzerland; 4 University of Basel, Basel, Switzerland; 5 World Health Organization, Phnom Penh, Cambodia; 6 Ministry of Health, 80, Samdech Penn Nouth Blvd. (289), Sangkat Boeungkak 2, Tuol Kork District, Phnom Penh, Cambodia; 7 RTI International, 701 13th St NW, Suite 750, Washington, DC USA; 8 RTI International, East Cornwallis Road, PO Box 12194, Research Triangle Park, NC USA; 9 World Health Organization Western Pacific Regional Office, Manila, Philippines; 10 Consultant on lymphatic filariasis, Tagore Nagar, Pondicherry, India

**Keywords:** Lymphatic filariasis, Elimination, Validation, Neglected tropical diseases, Cambodia

## Abstract

**Background:**

Endemicity of lymphatic filariasis (LF) in Cambodia was proven in 1956 when microfilariae were detected in mosquitos in the Kratié province. In 2001, an extensive study confirmed the presence of both *Brugia malayi* and *Wuchereria bancrofti* microfilariae*.* In 2003, the Ministry of Health established a national task force to develop policies and strategies for controlling and eliminating neglected tropical diseases (NTDs), with the goal of eliminating LF by 2015. This article summarizes the work accomplished to eliminate LF as a public health problem in Cambodia.

**Methods:**

The National Program to Eliminate Lymphatic Filariasis made excellent progress in the goal towards elimination due to strong collaboration between ministries, intensive supervision by national staff, and advocacy for mobilization of internal and external resources. Mass drug administration (MDA) with diethylcarbamazine citrate and albendazole was conducted in six implementation units, achieving > 70% epidemiological coverage for five consecutive rounds, from 2005 to 2009. In 2006, in 14 provinces, healthcare workers developed a line list of lymphedema and hydrocele patients, many of whom were > 40 years old and had been affected by LF for many years. The national program also trained healthcare workers and provincial and district staff in morbidity management and disability prevention, and designated health centers to provide care for lymphedema and acute attack. Two reference hospitals were designated to administer hydrocele surgery.

**Results:**

Effectiveness of MDA was proven with transmission assessment surveys. These found that less than 1% of school children had antigenemia in 2010, which fell to 0% in both 2013 and 2015. A separate survey in one province in 2015 using Brugia Rapid tests to test for LF antibody found one child positive among 1677 children. The list of chronic LF patients was most recently updated and confirmed in 2011–2012, with 32 lymphoedema patients and 17 hydrocele patients listed. All lymphedema patients had been trained on self-management and all hydrocele patients had been offered free surgery.

**Conclusions:**

Due to the success of the MDA and the development of health center capacity for patient care, along with benefits gained from socioeconomic improvements and other interventions against vector-borne diseases and NTDs, Cambodia was validated by the World Health Organization as achieving LF elimination as a public health problem in 2016.

**Electronic supplementary material:**

The online version of this article (10.1186/s40249-018-0394-7) contains supplementary material, which is available to authorized users.

## Multiligual abstract

Please see Additional file 1 for translations of the abstract into the five official working languages of the United Nations.

## Background

Lymphatic filariasis (LF), a major public health problem in many tropical and sub-tropical countries, is slated for elimination as a public health problem by 2020 by the World Health Organization (WHO). It is caused by three species of nematode filarial worms (*Wuchereria bancrofti*, *Brugia malayi*, and *B. timori*) and transmitted by mosquitoes. *Wuchereria bancrofti* is the predominant parasite and responsible for about 90% of the total LF infections. It causes clinical conditions of lymphedema and hydrocele, conditions that have significant social and economic consequences [1]. Prior to the launching of the Global Programme to Eliminate Lymphatic Filariasis (GPELF) in 2000, the disease was endemic in 80 countries, 1.1 billion people were living in known endemic areas, and 120 million people were infected [2]. Twenty-two countries in the WHO Western Pacific Region are endemic [1].

Development of new treatment strategies and the advent of new diagnostic tools in the 1980s and 1990s provided the impetus to strive for global elimination. The two pillars of GPELF are (a) transmission interruption through mass drug administration (MDA) of antifilarial drugs, and (b) alleviation of suffering in chronic patients through morbidity management and disease prevention (MMDP). Cambodia is among the first countries in the world to develop and institute an LF elimination program and successfully eliminate LF as a public health problem.

### Sociogeographic context

Cambodia is situated in Southeast Asia, bordered by the Gulf of Thailand, Thailand, Laos, and Vietnam. As of 2015, there are an estimated 15.6 million people living in the country, 21% of which live in urban areas [3]. The climate is tropical. The monsoon season lasts from May to November, and the dry season from December to April.

Cambodia is a lower-middle-income country, with an estimated gross national income per capita of US$ 1070 in 2015 [3]. Since 2000, Cambodia has witnessed tremendous economic growth, leading to a rise in consumption and a reduction in inequalities. Cambodia has also observed a significant improvement in the health status of the population; particularly in infant, child, and maternal mortality, as well as a continuing decline in HIV prevalence and deaths due to malaria [4].

### Building the LF elimination program

**Fig. 1:**
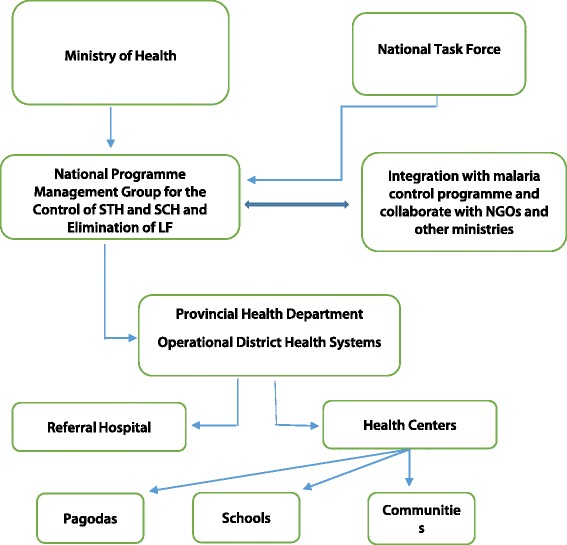
Structure of the Cambodian MoH and national task force for the control of STHs and schistosomiasis, and the elimination of LF

Before the launch of the GPELF in 2000, LF had been a low priority disease in Cambodia. In 2003, the Ministry of Health (MoH) established a national task force for the control of soil-transmitted helminthiases (STHs) and schistosomiasis, and the elimination of LF (see Fig. 1). Its major function is to develop the policies and strategies for neglected tropical disease (NTD) control and mobilization of resources. The members are drawn from the MoH and other ministries such as the Ministries of Education, Rural Development, and Water. Cambodia was one of the first countries to launch programs to address not only LF, but also two other major NTDs (STHs and schistosomiasis) simultaneously.

The national program established immediate goals to complete mapping of LF in the country, pilot LF MDA in the Ratanakiri province which had historical evidence of LF infection, and train all health personnel by 2004. The program also established intermediate goals of 50% LF MDA geographic coverage by 2005 and 100% by 2006, with final goals of interruption of transmission by 2010 and WHO validation of elimination by 2015.

Cambodia’s LF elimination program activities are all coordinated by the National Center for Parasitology, Entomology, and Malaria Control (CNM), under the direction of the national program manager. Provincial- or district-level health officers are responsible for supervising village health workers and community members who implement social mobilization and drug distribution activities.

Cambodia’s LF program is an excellent example of a lower-income country mobilizing human and financial resources, and forging partnerships to eliminate NTDs. Its background, implementation, and success are described in this paper.

## Methods

### Delineation of endemic provinces

#### History of LF

The evidence for prevalence of LF first emerged in 1956. Microfilariae were found in mosquitoes in khbal Trach Village, Sre Cha Commune, Snuol District, Kratié Province [5]. Studies undertaken in the 1990s provided concrete evidence of the species of filarial parasites prevalent in Cambodia. In 1997, the microfilariae found in the night blood samples of inhabitants of Tiruom Srok Taveng Village, Taveng District, Stung Treng Province were diagnosed as *W. bancrofti* [6]. In the same year, further evidence for *W. bancrofti* prevalence in the Stung Treng Province was recorded; some people subjected to the immunochromatographic test (ICT), which detects circulating filarial antigenemia of *W. bancrofti*, in Sdao Village, Stung Treng District, Stung Treng Province, showed a positive reaction [5, 7]. An extensive study assessed the burden of LF in northeast Cambodia in February–April 2001, in which different LF burden estimation techniques such as a key-informant questionnaire, clinical examination, microfilaria surveys, and antigenemia surveys were compared. In this study, conducted in the Ratanakiri Province, both *B. malayi* (0.81%) and *W. bancrofti* (0.32%) microfilariae were found in night blood samples of tested subjects (*n* = 618) [5]. Thus, while more than one report confirms the prevalence of *W. bancrofti*, the Ratanakiri study indicated a co-prevalence of *B. malayi* and *W. bancrofti*. However, there are no reports or evidence that suggest prevalence of *B. malayi* in animals in Ratanakiri.

While it was recorded that both *W. bancrofti* and *B. malayi* were prevalent in the country, no LF vector studies could be undertaken due to paucity of trained personnel and information on the distribution of LF. The disease was also considered of low public health importance and of low priority. Hence, it is unclear as to which species of mosquitoes are involved in the transmission of LF. Cambodia has a rich fauna of *Anopheles* species, some of which are involved in the transmission of malaria [8]. Several species of *Anopheles* mosquitoes are involved in the transmission of bancroftian and brugian filariasis in the Southeast Asia region [9]. Involvement of one or more species of *Anopheles* mosquitoes in the transmission of *W. bancrofti* and/or *B. malayi* in Cambodia may be a possibility.

The history of LF clinical disease is relatively recent in Cambodia. A person with filarial elephantiasis was reported from Stung Treng in 1995 by Medecins Sans Frontières [6]. Subsequently, the MoH made considerable efforts to estimate the burden of chronic disease in different provinces, as part of the LF mapping exercise under the LF elimination program. In 2001, in order to estimate the burden of LF and clinical cases, the CNM conducted a rapid assessment using a simple questionnaire sent to three key informants in all villages of 13 provinces. The questionnaires collected the following information: 1) name and address of the key informant; 2) five most prevalent diseases in the village; 3) presence of individuals with swollen legs; and 4) presence of individuals with swollen scrotums (for numbers 3 and 4, pictures were provided along with the questionnaire). Chronic patients were estimated based on the responses provided by the key informants from the villages, however, this method tended to overestimate the burden of lymphedema and hydrocele patients since key informants were not medically trained in diagnosing lymphedema and hydrocele [5] .

#### Mapping

To overcome the bias of rapid assessments, the CNM decided to implement antigenemia surveys in all 25 provinces in 2001–2002. In each province, five villages were selected randomly from the list of all villages. In the selected villages, the provincial health personnel with the support of the district-level and health center staff carried out the survey. In each village, 50 adults were randomly selected and assessed for antigenemia using ICTs. From the results of the surveys, four northeastern provinces (Ratanakiri, Stung Treng, Siem Reap, and Preah Vihear) were identified as having antigenemia-positive individuals. In addition, a research study in Ratanakiri, Stung Treng, Preah Vihear, and Mondulkiri was implemented in 2001 to compare different survey methods. In each of the 21 districts in these four provinces, 3–9 villages were assessed for antigenemia and microfilaremia prevalence, and 243 to 321 people per district were blood tested for *W. bancrofti* antigenemia using ICTs and microfilaremia using night blood smears [5].

**Fig. 2:**
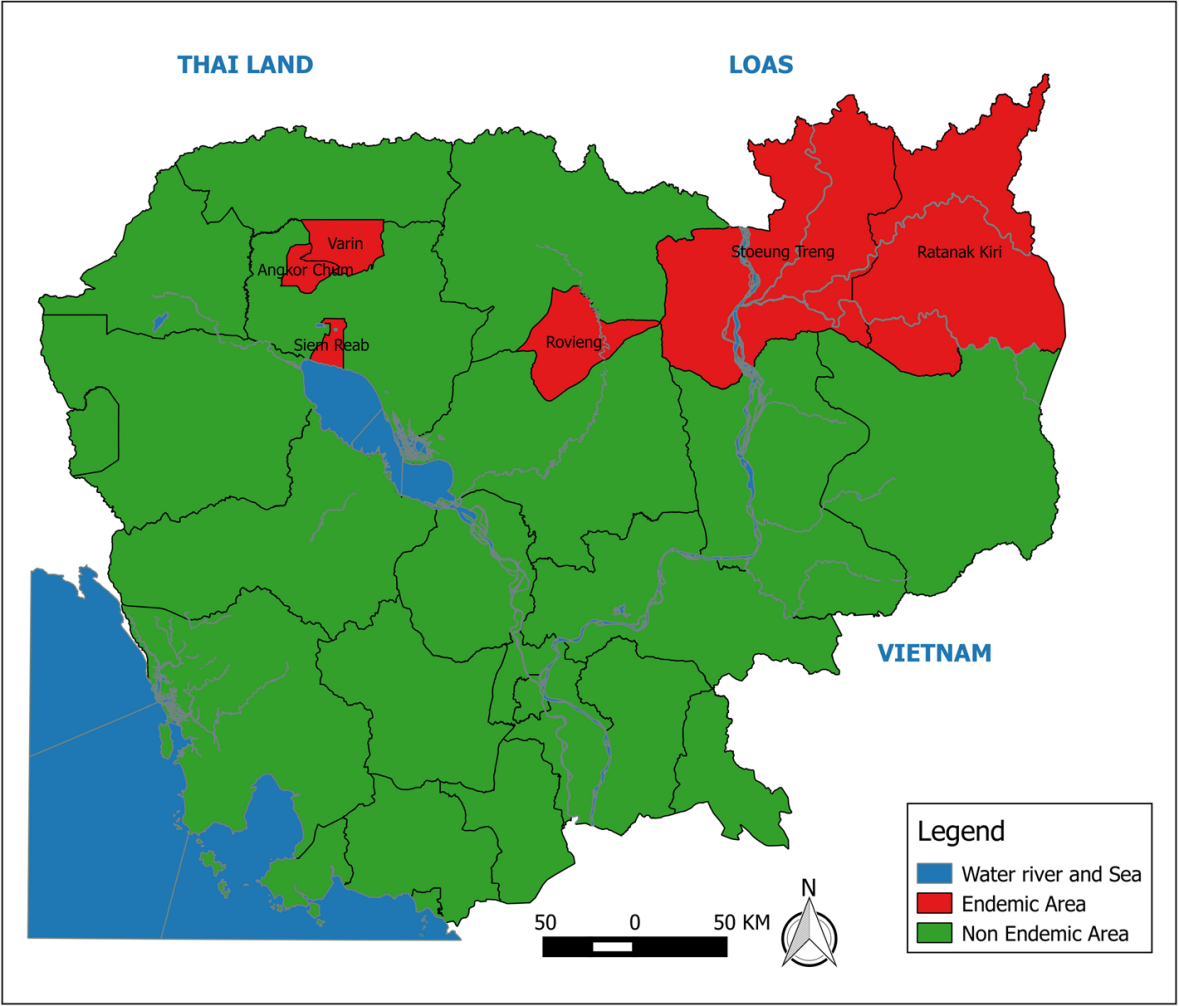
Map of LF endemic provinces in Cambodia

On the basis of the presence of clinical cases, microfilaria prevalence and an assessment of antigenemia prevalence at the province and district levels (2000–2004), two provinces were declared entirely endemic, Ratanakiri and Strung Treng, and four districts in two provinces were classified as endemic due to the focalized nature of LF (Rovieng in Preah Vihear province, and Varin, Angkor Chum, and Siem Reap in Siem Reap province) (see Fig. 2). Although the antigenemia prevalence was < 1.0% in some districts, the program decided to take a conservative approach and classify any district with positive cases as endemic, as well as implement MDA in order to eliminate infection even in low endemic foci. Based on this criterion, the CNM designated both the province and district as the implementation unit.

### Implementation of the MDA program

#### Human resources

The CNM guided each province to build a core team of 15–20 personnel to plan and effectively implement the MDA program. These personnel were then organized into 4–5 teams for implementation. In order to deal with any adverse events during the MDA and provide close guidance and direction for drug distribution activities, the national program manager and other CNM personnel worked closely with drug distribution teams and spent 2–3 months in the endemic provinces during each round of MDA.

#### Training

The personnel involved in drug distribution were trained by the CNM and provincial health department staff prior to each round of MDA. The training focused on the social and economic effects of LF, the objectives and rationale of the MDA program, importance of high treatment coverage, and how to address adverse events due to treatment. The numbers of personnel trained are summarized in Table 1.

**Table 1:** Training of health personnel for the LF elimination program in Cambodia

Administration level	Training on transmission interruption				Training on MMDP		
	Number of courses organized/ number of staff trained				Number of courses organized/ number of staff trained		
	2006	2007	2009	2010	2007	2009	2010
Provincial	2/158	2/150	2/50	2/45	2/100	2/40	2/20
District	4/50	4/40	3/30	4/15	4/200	3/20	3/10

#### Social mobilization

The communities were informed of the drug distribution 1–2 days prior through the use of posters, loud speakers, and leaflets. The head of the village played a key role in ensuring the participation of the entire village. Health personnel also played a role in social mobilization by informing people about the risks of infection, and the social and economic impact LF can have on communities.

#### Drug distribution

Albendazole (ALB) was provided by GlaxoSmithKline through the WHO donation program and diethylcarbamazine citrate (DEC) was procured locally by the MoH. The provinces submitted their application for drugs through an internal electronic system, which triggered the shipping process of the requested medicine from the central storage system to the distribution sites. The teams disbursed the drugs from central locations, such as pagodas, schools, and community halls. If the people were not able to come to the central location to receive the drug, the teams took the drugs to their homes to impart treatment. The local healthcare workers and other personnel involved in the drug distribution were given incentives and allowances to meet the costs of food, transportation, and accommodation. Realizing the benefits of the program, the health personnel implemented the drug distribution program with a great deal of enthusiasm and commitment.

Drug distribution was staggered, with only two implementation units conducting MDA at a time in order for the CNM to participate in and directly observe treatment in all implementation units. The teams and CNM personnel worked with village healthcare workers and other governmental staff such as teachers to implement drug distribution at the community level. The teams completed drug distribution in a cluster of villages and then moved to the next cluster in each province. The teams required approximately 1 month’s time to complete drug distribution in a province. The presence of central personnel and their participation in drug distribution activities provided good advocacy, supervision, and goodwill for the program.

#### Adverse events

Incidence of adverse events was assessed within 2 hours of implementing the MDA in 2006. Village healthcare volunteers were asked to call the health center if a serious adverse event occurred. If an event was reported, the health center completed the WHO serious adverse events form, which was submitted to the team implementing the MDA, and the patient suffering the adverse event was transferred to the referral hospital.

Dizziness and nausea were the major events observed. Mild and moderate adverse reactions were easily managed and no serious adverse event was reported. The affected people were advised to follow traditional treatment methods such as drinking coconut water or tea. Medicines were used very rarely to deal with adverse events.

### Morbidity management and disease prevention (MMDP)

After establishing the MDA program in the endemic areas, in order to achieve interruption of transmission, the CNM turned its attention to MMDP activities, the second pillar of the LF elimination program. In 2006, in 14 provinces, a list of patients was prepared by the healthcare workers, who went door to door to examine suspected patients for lymphedema or hydrocele. Forty cases of lymphedema and 18 cases of hydrocele were found. Most of the patients were > 40 years old and had been affected for many years. The CNM designated the health center as the health facility to provide services for lymphedema and acute adenolymphangitis, or acute attack management services, with complicated cases referred to provincial-level health facilities. For hydrocele surgery, given the limited capacity of hospitals in the endemic districts, the program designated two reference hospitals for surgery in Kampong Cham and Phnom Penh.

## Results

### Program coverage

The first round of MDA was implemented in 2005 and the target population was 434 999. In total, five rounds of MDA were implemented consecutively from 2005 to 2009. In all implementation units, treatment coverage exceeded the ‘effective level’ of 65% set by the WHO during every round of MDA. The lowest average annual coverage reported was 67% in 2005 (see Table 2).

**Table 2:** Summary of national MDA data by implementation unit, by year, Cambodia

Implementation unit	Population (2008 census)	MDA coverage (% of the total population treated) by year						
		2005	2006		2007	2008	2009	
		Reported	Reported	Surveyed	Reported	Reported	Reported	Surveyed
Rovieng	35 663	76	84	77	79	78	83	82
Ratanakiri	150 466	76	79	75	86	89	81	85
Angkor Chum	53 768	73	76	77	82	81	88	86
Siem Reap	174 265	77	78	72	80	82	84	73
Varin	32 026	77	81	82	80	77	78	84
Stung Treng	111 671	67	77	73	80	80	90	74

In order to verify if the reported treatment coverage was robust, independent treatment coverage surveys were conducted in all implementation units after MDA in 2006 and 2009. Those staff members from CNM and provinces who were not associated with the LF MDA program were drafted to conduct the coverage surveys. These surveys were conducted following the protocol recommended by the WHO [10]. In each implementation unit, 30 villages were sampled to assess the MDA treatment coverage, with 10 randomly selected houses per village sampled. Completed questionnaires were sent to the CNM, where the data were compiled and treatment coverage was calculated. The differences between reported treatment coverage and surveyed treatment coverage were minimal (see Table 2).

### Monitoring and evaluation

Despite the relatively low baseline prevalence of LF in the mapping sites, the country implemented a robust monitoring and evaluation plan to monitor the progress of the program. Two sentinel sites and two spot check sites were selected per implementation unit. The sentinel sites were purposefully chosen from those with the presence of either microfilariae or lymphedema or hydrocele patients, while the spot check sites were chosen randomly.

In each sentinel and spot check site, all the households were listed and a sample of 60–120 households were randomly selected during different surveys. All members of the selected households aged > 5 years were assessed for LF infection. Blood samples were collected at night between 20.00 and 23.00 h. From each person in the selected households, two thick blood smears of 20 μl each were collected using disposable blood lancets. The blood smears were dried overnight and stained the next day using Giemsa stain. The stained blood smears were examined in the village itself, allowing for the results to be immediately available. In the sentinel sites, microfilaria surveys were carried out during the baseline year (2005) and interim years (2006, 2007, and 2008), and after the final round of MDA (2009). In the spot check sites, the surveys were carried out in 2007, 2008, and 2009.

The baseline microfilaria prevalence in the sentinel sites ranged from 0.00% to 1.80% in the six implementation units. It fell to 0% in all implementation units after two rounds of MDA. In the spot check sites, the microfilaria prevalence was found to be 0% in all surveys. Though the microfilaria prevalence declined to 0% after two rounds of MDA, the third, fourth, and fifth rounds of MDA were implemented as effectively as possible, which was evident from the high treatment coverage rates.

### Stopping the MDA program

The fifth round of MDA was implemented in 2009 and surveys to determine whether MDA could be stopped were implemented from October to November 2010. The CNM worked in close collaboration with the provincial health departments and provincial education department to conduct the surveys. As the WHO guidance on administering transmission assessment surveys (TASs) was not ready in 2010, a sampling protocol was developed with the support of a technical expert to conduct the stopping MDA surveys.

The protocol advised sampling 42 schools in the six implementation units and 900 primary school children aged 6–10 per unit. The implementation units were divided into two categories: implementation units with relatively higher baseline microfilaria prevalence, which also happened to be larger, i.e. provinces (Ratanakiri and Stung Treng), and implementation units with relatively lower microfilaria prevalence, which happened to be smaller, i.e. districts (Siem Reap, Angkor Chum, Varin, and Rovieng). In the former category, 27 schools were randomly selected and in the latter category 15 schools were randomly selected. Within the selected schools, the required sample was obtained by using a sampling interval. A few days prior to the visit of survey teams, the provincial and district health departments informed the selected primary schools of the survey team’s visit, dates of activity, survey procedures, and the need to sensitize the community and set up necessary infrastructure in the schools. The schools extended all support to surveys: informed parents about the survey and a ‘collective consent’ was obtained from the villages’ communities. Any parent who did not wish his/her child to be blood examined was allowed to withdraw the child from the survey.

The implementation unit was also determined to be the evaluation unit for stopping MDA surveys and the six implementation units were designated as six evaluation units. A total of 5400 children from 42 schools in six evaluation units were tested for *W. bancrofti* antigenemia using ICT. The number of positive children per evaluation unit was 1–6 (see Table 3). The antigenemia prevalence in different evaluation units ranged from 0.11% to 0.67%, below a conservative prevalence level of < 1.00% to stop MDA. Hence, the program decided to stop MDA in all implementation units. All children found with antigenemia were treated with a single dose of DEC + ALB.

**Table 3:** Results of the stopping MDA surveys, and TAS 2 and TAS 3

Province	Evaluation unit	Stopping MDA surveys (2010)		TAS 2 (2013)			TAS 3 (2015)		
		Sample	Positive (%)	Sample	Positive (%)	Critical cut-off value	Sample	Positive (%)	Critical cut- off value
Preah Vihear	Rovieng	900	2 (0.22)	1750	0	16	1531	0	16
Ratanakiri	Ratanakiri	900	4 (0.44)	1805	0	18	1824	0	18
Siem Reap	Angkor Chum	900	1 (0.11)	1575	0	18	1728	0	18
	Siem Reap	900	6 (0.67)						
	Varin	900	2 (0.22)						
Stung Treng	Stung Treng	900	3 (0.33)	1755	0	18	1582	0	18

### Post-MDA surveillance

The post-MDA surveillance surveys were based on 2011 WHO guidance, which recommends implementation of TASs 2–3 years (TAS 2) and 4–6 years (TAS 3) after stopping MDA.

TAS 2 was conducted in 2013, and as the enrolment rate exceeded 75%, school-based TAS involving children in grades 1 and 2 was decided upon. The cluster sampling methodology was followed and the number of schools and children to be sampled and the sampling interval were determined using the transmission assessment survey sample builder Excel tool (www.ntdsupport.org/resources). For the purposes of TAS 2, the six implementation units were reorganized into four evaluation units, combining the three district implementation units of Siem Reap province into one evaluation unit, and leaving the other three implementation units as evaluation units. The number of children sampled in the four evaluation units ranged from 1575 to 1805. None of the sampled children in any evaluation unit was positive for antigenemia and the antigenemia prevalence was 0% (see Table 3).

TAS 3 was conducted in 2015, two years after TAS 2. The methodology followed for TAS 3 was similar to that followed for TAS 2. In the four evaluation units, 1531 to 1824 children were sampled for the assessment of *W. bancrofti* antigen in children. A total of 6665 children were examined in the four evaluation units and all children were found to be negative for this antigen (see Table 3).

These results suggest that the total transmission interruption status of evaluation units, indicated by TAS 2, continues to be sustained. In the Ratanakiri evaluation unit, a separate TAS 3 was undertaken for assessment of *B. malayi* antibody, given historical evidence of *B. malayi* transmission [5]. In this survey, 1677 children were tested using the Brugia Rapid test (Reszon Diagnostics, Selangor, Malaysia) with a critical cut-off of 18, and one child was found positive (results not included in Table 3).

### MMDP patient care

Lymphoedema patients were given training by healthcare workers on maintaining leg hygiene and avoiding infections, following WHO guidance [11]. Patients were also trained on how to cope and manage acute adenolymphangitis episodes. They were provided with patient self-care guides and morbidity management kits containing soap, antibiotics, paracetamol, and gauze cloth. These kits were provided each year during the MDA implementation, from 2005 to 2009. Additionally, patients were advised to buy the components of the kits themselves, which allowed self-treatment to continue post-MDA. These activities led to a good rapport between the patients and healthcare workers. Sixteen follow-up training courses were held from 2007 to 2010 for provincial and district health center staff on the role and importance of MMDP and the methods of morbidity management, including teaching patients self-care of lymphedema, and diagnosing and treating acute attacks (see Table 1).

Significant efforts were also made to address the issue of surgical intervention for hydrocele patients. No hydrocele or other surgeries are performed in the endemic provinces due to limited infrastructure and expertise. The program tried to convince hydrocele patients to travel to the Kampong Cham Provincial Hospital in Kampong Cham province or Calmette Hospital in Phnom Penh to undergo surgery, by offering free surgeries and support to patients’ costs of travel. However, the patients were not willing to travel and undergo surgeries, as they were of advanced aged and feared post-surgery complications and long recuperation time.

The list of chronic LF patients was most recently updated and confirmed in 2011–2012, with 32 lymphoedema and 17 hydrocele patients listed. The data suggest that the chronic LF disease burden is not significant and the burden over the years has been decreasing.

## Discussion

### Role of the government

Cambodia was able to achieve remarkable results to eliminate LF in the endemic provinces due to the commitment of the government and effective implementation of MDA, monitoring and evaluation, and surveillance activities. Although the endemic provinces are forested and remote areas, MDA was successfully implemented through advocacy, sensitization of various departments, active participation of provincial- and central-level program personnel in MDA activities, and financial and related support from partners and stakeholders.

### Partnership

The program built a strong partnership with international agencies with the objective of effectively implementing the program. The major partners include the WHO, the United States Agency for International Development, FHI 360, RTI International, and the Cambodia Second Health Sector Support Program. The partners supported the program by providing financial and technical assistance, as well as by providing training to implement the MDA program.

### Complementary data

The four provinces endemic for LF have also been highly endemic for malaria. Distribution of free long-lasting insecticide nets (LLINs) began in 2000. The proportion of the high-risk population protected with LLINs reached 40% by 2009 and close to 100% by 2012 [8]. These nets provide protection against malaria vectors and, to some extent, against vectors of other vector-borne diseases, including LF. There has been a gradual socioeconomic improvement in the provinces, as well as a very robust MDA program against STHs in the entire country. The STH program distributes ALB or mebendazole to preschool and school children, treating both enrolled as well as non-enrolled children, and also treats a proportion of women of childbearing age. The program targeted 8.38 million school children in 2016, 2.71 million preschool children, and nearly four million women of childbearing age [12]. Together, these factors make resurgence of LF in the provinces very unlikely.

### Post-validation surveillance

In 2012, the Cambodia MoH implemented a serological survey among women aged 15–39 to assess immunity to various diseases, including tetanus and rubella [13]. As part of this survey, antibody responses to a variety of parasitic infections, including *W. bancrofti*, were measured by multiplex bead assay*.* The results found residual antibody reactivity in the North region, which includes the LF endemic areas, and an absence of activity in non-MDA areas. Building on the success of that research as a platform to collect LF data from throughout the country, the program intends to implement a post-validation surveillance system that will be integrated into routine population-based surveys or ongoing collection of other surveillance data. In 2017, with support from the WHO, the CNM will be working with the surveillance section of the MoH to determine a sustainable strategy, including what type of diagnostic tests, sampling methodology, and sample population will be used. Sensitive diagnostic tools such as serological and molecular tests need to be made available at the reference laboratory at the central level.

## Conclusions

### LF elimination dossier

In 2015, the MoH prepared its dossier documenting the elimination of LF as a public health problem. It included data on LF mapping in the country to determine endemic provinces, the establishment of the national LF elimination program and its robust implementation of MDA, data collected from the sentinel and spot check sites, results from the stopping MDA surveys, as well as a summary of post-MDA surveillance activities (TASs 2 and 3). The dossier also included information on how the program collected the numbers of chronic LF cases and how the health system is training, treating, and monitoring those cases to ensure they are receiving the care they need. The dossier was submitted for approval to the MoH and validated by the Regional Dossier Review Group of WHO’s Western Pacific Regional Office. In June 2016, WHO headquarters officially acknowledged that elimination of as a public health problem was achieved in Cambodia.

## Additional file

Additional file 1: Multiligual abstract in the five official working languages of the United Nations. (PDF 628 kb)

## Abbreviations

ALB: Albendazole
CNM: National center for parasitology, entomology, and malaria control
DEC: Diethylcarbamazine citrate
GPELF: Global programme to eliminate lymphatic filariasis
ICT: Immunochromatographic test
LF: Lymphatic filariasis
LLIN: Long-lasting insecticide net
MDA: Mass drug administration
MMDP: Morbidity management and disease prevention
MoH: Ministry of Health
NTD: Neglected tropical disease
STH: Soil-transmitted helminth
TAS: Transmission assessment survey
WHO: World Health Organization

## References

[1] World Health Organization (2016). Global programme to eliminate lymphatic filariasis: progress report, 2015. Wkly Epidemiol Rec.

[2] World Health Organization (2001). Lymphatic Filariasis. Wkly Epidemiol Rec.

[3] World Bank (2017). World development indicators 2017.

[4] Cambodia: WHO statistical profile. World Health Organization: Website: http://www.who.int/gho/countries/khm.pdf. Accessed 9 Feb 2018.

[5] Leang R, Socheat D, Bin B, Bunkea T, Odermatt P (2004). Assessment of disease and infection of lymphatic filariasis in Northeastern Cambodia. Tropical Med Int Health.

[6] Urbani C (1997). Control of schistosomiasis and other Helminthiasis in Cambodia.

[7] World Health Organization Western Pacific Regional Office. First mekong-plus programme managers’ Workshop on Lymphatic Filariasis and other Helminthiasis. Manila: WHO; 2009.

[8] Cambodia Malaria Country Profile. [http://www.who.int/malaria/publications/country-profiles/profile_khm_en.pdf?ua=1]. Accessed 9 Feb 2018.

[9] Ramalingam S, Guptavanij P, Harinasuta C, Sandosham A, Zaman V (1968). The vectors of W. bancrofti and B. malayi in South-east Asia. Proceedings of a seminar on filariasis and immunology of Parasitic Infections.

[10] World Health Organization. Global programme to eliminate lymphatic filariasis: monitoring and epidemiological assessment of mass drug administration. Geneva: WHO; 2011.

[11] World Health Organization (2003). Training module on community home-based prevention of disability due to lymphatic filariasis.

[12] Ministry of Health of Cambodia. Annual report of the national center for parasitology, entomology and malaria control. Phnom Penh: Cambodia Ministry of Health; 2015.

[13] Priest JW, Jenks MH, Moss DM, Mao B, Buth S, Udhayakumar V, Gregory CJ, Huy R, Muth S (2016). Integration of multiplex bead assays for parasitic diseases into a national, population-based serosurvey of women 15- 39 years of age in Cambodia.

