# Supplementary material for: How elimination of lymphatic filariasis as a public health problem in the Kingdom of Cambodia was achieved
Source: Infect Dis Poverty. 2018 Feb 20;7:15. doi: 10.1186/s40249-018-0394-7 (PMC5819284; doi:10.1186/s40249-018-0394-7)
Supplement: Supplementary file 1 — Multiligual abstract in the five official working languages of the United Nations. (PDF 628 kb) [file 40249_2018_394_MOESM1_ESM.pdf]

Translation of the abstract into the five official working languages of the United Nations

كيف تم القضاء على داء الفيلاريات اللمفاوية كمشكلة صحية عامة في مملكة كمبوديا

فيراك خيو، فاندين أور، تشاكدا تيب، بيتر أودرمات، ريكو تسويوكا، منج تشور شار، مولي أ برادي، جوشوا سيدويل، آيا ياجيما، ريكول هوي، كابا د رميا و سينون موث

ملخص

خلفية: ثبت توطن داء الفيلاريات اللمفاوي في كمبوديا في عام 1956 عندما تم الكشف عن الميكروفيلاريا في البعوض في مقاطعة كراتيه. في عام 2001، أكدت دراسة واسعة وجود كل من اليرقات الخيطية الدقيقة البروجية الملاوية والفخرية بنكروفتية. وفي عام 2003، أنشأت وزارة الصحة فرقة عمل وطنية لوضع سياسات واستراتيجيات لمكافحة أمراض المناطق المدارية المهملة والقضاء عليها، بهدف القضاء على داء الفيلاريات اللمفاوي بحلول عام 2015. وتلخص هذه المادة العمل المنجز للقضاء على داء الفيلاريات اللمفاوي كمشكلة صحية عامة في كمبوديا.

الأساليب: حقق البرنامج الوطني للقضاء على داء الفيلاريات اللمفاوي تقدما ممتازا في الهدف نحو القضاء على المرض بسبب التعاون القوي بين الوزارات، والإشراف المكثف من جانب الموظفين الوطنيين، والدعوة لتعبئة الموارد الداخلية والخارجية .وقد تم توزيع دوائي سيترات ديثيلكاربامازين وألبيندازول على نطاق جماهيري في ست وحدات تنفيذ، وتحقيق تغطية وبائية أقل من 70٪ لخمسة جولات متتالية، من عام 2005 إلى عام 2009. في عام 2006، في 14 مقاطعة، وضع العاملون في مجال الرعاية الصحية قائمة خطية لمرضى الوذمة اللمفية والقيلة المائية، وكان العديد منهم عمره أكبر من 40 سنة، وتضرر من داء الفيلاريات اللمفاوية لسنوات عديدة. وقام البرنامج الوطني أيضا بتدريب العاملين في مجال الرعاية الصحية وموظفي المقاطعات والمقاطعات في مجال إدارة الاعتلال والوقاية من الإعاقة، كما عين مراكز صحية لتوفير الرعاية للوذمة الليمفاوية والهجوم الحاد. تم تعيين مستشفيين مرجعيين لإدارة إجراء عمليات القيلة المائية.

النتائج: أثبتت فعالية توزيع الأدوية على نطاق جماهيري في الدراسات الاستقصائية لتقييم الانتقال. وجد أن أقل من 1٪ من أطفال المدارس لديهم مستضدات في الدم في عام 2010، والتي انخفضت إلى 0٪ في كل من 2013 و2015. مسح منفصل في مقاطعة واحدة في عام 2015 باستخدام اختبارات بروجيا السريعة لاختبار الأجسام المضادة اللمفاوية داء الفيلاريات وجدت طفل واحد إيجابي بين 1677 طفلا. تم تحديث قائمة مرضى داء الفيلاريات اللمفاوي المزمن مؤخرا وتم تأكيدها في 2011-2012، مع 32 مريضا وذمة لمفية و17 مريض القيلة المائية. تم تدريب جميع مرضى الوذمة اللمفاوية على الإدارة الذاتية، وتم إجراء جراحة لجميع المرضى الذين يعانون من القيلة المائية مجانا.

الاستنتاجات: نظرا لنجاح توزيع الدواء على نطاق جماهيري وتطوير قدرة المركز الصحي على رعاية المرضى، إلى جانب الفوائد المكتسبة من التحسينات الاجتماعية والاقتصادية والتدخلات الأخرى ضد الأمراض المنقولة بالنواقل والأمراض المدارية المهملة، أجيزت كمبوديا من قبل منظمة الصحة العالمية كما تحقيق القضاء على داء الفيلاريات اللمفاوية كمشكلة الصحة العامة في عام 2016.

Translated from English version into Arabic by Mahmoud Sami, through

TRANSLATORS WITHOUT BORDERS

柬埔寨淋巴丝虫病消除策略

Virak Khieu, Vandine Or , Chhakda Tep , Peter Odermatt , Reiko Tsuyuoka , Meng Chuor Char , Molly A Brady , Joshua Sidwell, Aya Yajima, Rekol Huy, Kapa D Ramaiah and Sinuon Muth

摘要

引言：1956 年，科学家在 Kratie 省的蚊子体内发现了微丝蚴，以此证明了柬埔寨的淋巴丝虫病(LF)的地方性流行。2001 年，一项广泛的研究证实了马来亚和乌尔切里亚(bancrofti)微丝蚴的存在。2003 年，柬埔寨卫生部成立了一个国家工作队，制定了控制和消除被忽视的热带病的政策和战略，目标是 2015 年消除被忽视的热带病。本文概述了柬埔寨消除 LF 这一公共卫生问题的策略和行动。

方法：由于各部之间的密切合作，国家工作人员的有力监督和宣传动员内部和外部资源，消除淋巴丝虫病国家项目取得了良好的进展。2005 年至 2009 年间， 6 家单位采用枸橼酸二乙碳酰嗪和阿苯达唑开展了大规模群体服药(MDA)，并连续开展 5 轮，流行病学覆盖率达到 70%以上。2006 年，14 个省的医护人员确定了一系列的淋巴水肿和水囊肿患者的名单，患

者大多在 40 岁以上，多年来一直受到 LF 的折磨。国家项目还针对发病率管理和残疾预防对护理工作者和省及地区工作人员进行培训，并指定护理中心为淋巴水肿和急性发作提供治疗；指定两家参比医院进行阴囊积水手术。
结果：本研究通过对传播评估的调查证实了 MDA 的有效性。2010 年，发现在有不到 1%的儿童有抗源性贫血症， 2013 年和 2015 年降至 0%。2015 年，在一个省的另一项调查中，使用 Brugia 快速测试对 LF 抗体进行检测，发现 1 677 名儿童中只有一个阳性。慢性 LF 患者的名单进行更新并在 2011 - 2012 年复核，其中有 32 名淋巴水肿患者和 17 名水囊肿患者。所有的淋巴水肿患者都接受了自我管理的训练，并且所有的水囊肿患者都接受了免费手术。
结论：由于 MDA 的成功和医疗中心对病人护理能力的提高，同时获益于社会经济的进步以及对其它媒介传播疾病和被忽视热带病的干预，世界卫生组织确认柬埔寨于 2016 年消除除淋巴丝虫病。

Translated from English version into Chinese by Xin-Yu Feng, through

CHINESE CENTER FOR DISEASE CONTROL AND PREVENTION
NATIONAL INSTITUTE OF PARASITIC DISEASES

Élimination de la filariose lymphatique en tant que problème de santé publique au royaume du Cambodge

Virak Khieu, Vandine Or , Chhakda Tep , Peter Odermatt , Reiko Tsuyuoka , Meng Chuor Char , Molly A Brady , Joshua Sidwell, Aya Yajima, Rekol Huy, Kapa D Ramaiah et Sinuon Muth

Résumé

Introduction : Le caractère endémique de la filariose lymphatique (FL) au Cambodge a été prouvé en 1956 suite à la détection de microfilaires dans des moustiques, dans la province de Kratie. En 2001, une étude à grande échelle a confirmé la présence des microfilaires *Brugia malayi* et *Wuchereria bancrofti.* En 2003, le Ministère de la Santé a mis en place un programme national visant à développer des mesures et stratégies de contrôle et d'élimination des maladies tropicales négligées (MTN), fixant comme objectif l'élimination de la FL d'ici 2015. Cet article résume les efforts fournis pour éliminer la FL en tant que problème de santé publique au Cambodge.
Méthode : Le programme national d'élimination de la filariose lymphatique a donné d'excellents résultats en termes d'élimination de la maladie grâce à une collaboration forte entre les ministères, à la surveillance intensive exercée par le personnel employé au niveau national et à des actions en faveur de la mobilisation des ressources internes et externes. Un traitement médicamenteux de masse (TMM), par l'administration de citrate de diéthylcarbamazine et d'albendazole, a été effectué dans six unités de mise en œuvre, atteignant une couverture épidémiologique supérieure à 70 % sur cinq campagnes consécutives de 2005 à 2009. En 2006, dans 14 provinces, des professionnels de la santé ont établi une liste des patients présentant un lymphœdème ou une hydrocèle ; parmi ceux-ci figuraient de nombreux patients de plus de 40 ans infestés par la FL depuis de nombreuses années. Ce programme national a également permis de former des professionnels de la santé et du personnel employé par la province ou le district à la prise en charge de la morbidité et à la prévention des incapacités ; des centres de soins désignés ont également été formés au traitement du lymphœdème et aux crises aiguës qui y sont associées. Deux hôpitaux de référence ont été désignés pour le traitement chirurgical de l'hydrocèle.
Résultats : L'efficacité du TMM a été mise en évidence par les enquêtes d'évaluation de la transmission. D'après celles-ci, moins de 1 % des enfants scolarisés présentait une antigénémie en 2010, un chiffre tombé à 0 % en 2013 et en 2015. Lors d'une enquête menée séparément dans une province en 2015, à l'aide des tests *Brugia Rapid*™ permettant de détecter les anticorps de la FL, un enfant sur 1677 présentait des anticorps. La liste des patients chroniques a très récemment été mise à jour et validée sur 2011 – 2012, avec 32 patients souffrant de lymphœdème et 17 souffrant d'hydrocèle. Tous les patients souffrant de lymphœdème avaient été formés à l'auto-prise en charge de leur maladie et tous les patients souffrant d'hydrocèle avaient été opérés gratuitement.
Conclusion : Grâce au succès du TMM et au développement des compétences de prise en charge des patients dans les centres de soins, aux bénéfices issus d'améliorations socio-économiques et issus d'autres interventions de lutte contre les maladies à transmission vectorielle et les MTN,

l’Organisation Mondiale de la Santé a validé l’élimination de la FL en tant que problème de santé publique au Cambodge.

Translated from English version into French by Anne Boudrot, through

Как было достигнуто устранение лимфатического филяриатоза в качестве проблемы общественного здравоохранения в Королевстве Камбоджа

Вирак Хиеу, Вандин Ор, Чхакда Теп, Питер Одерматт, Рейко Цуюока, Менг Чуор Чар, Молли А. Брэйди, Джошуа Сидуэлл, Айа Ядзима, Рекол Хуй, Капа Д. Рамайа и Синуон Мут

Реферат

Предпосылки: Эндемичность лимфатического филяриатоза (ЛФ) в Камбодже была доказана в 1956 г., когда микрофилярии были обнаружены у москитов в провинции Кратьэх. В 2001 г. в интенсивном исследовании было подтверждено наличие обеих микрофилярий *Brugia malayi* и *Wuchereria bancrofti.* В 2003 г. Министерство здравоохранения учредило национальную рабочую группу для разработки политики и стратегии по сдерживанию и устранению забытых тропических болезней (ЗТБ) с целью устранить ЛФ к 2015 г. В настоящей статье сводится опыт работы по устранению ЛФ в качестве проблемы общественного здравоохранения в Камбодже.

Методы: Национальной программе по устранению лимфатического филяриатоза удалось значительно продвинуться на пути к его устранению благодаря тесному сотрудничеству между министерствами, интенсивному вниманию со стороны национального рабочего коллектива и работе по привлечению внутренних и внешних ресурсов. В шести подразделениях, занимавшихся ее реализацией, проводилось массовое применение лекарственных средств (МПЛ) в виде цитрата диэтилкарбамазина и альбендазола и после пяти циклов подряд с 2005 по 2009 гг. был достигнут эпидемиологический охват больше 70%. В 2006 г. в 14-ти провинциях работниками здравоохранения был создан линейный список пациентов с лимфедемой и гидроцеле, многие их которых были старше 40 лет и страдали ЛФ многие годы. В рамках национальной программы проводилось также обучение работников здравоохранения и работников из провинций и регионов по контролю заболеваемости и предотвращению инвалидности, а в специализированных центрах здоровья обеспечивалось лечение лимфедемы и острых приступов. Для проведения хирургии гидроцеле были выделены две опорные больницы.

Результаты: В оценочных исследованиях переноса была доказана эффективность МПЛ. В них найдено, что меньше 1% школьников имели антигенемию в 2010 г., и это значение упало до 0% и в 2013, и в 2015 гг. В отдельном исследовании в одной из провинций в 2015 г. с использованием экспресс-тестов Brugia для проверки на антитела к ЛФ положительный результат был получен у одного ребенка из 1667 детей. Список хронических больных ЛФ в последний раз обновлялся и подтверждался в 2011-2012 гг., тогда в нем было 32 пациента с лимфедемой и 17 пациентов с гидроцеле. Все пациенты с лимфедемой были обучены оказывать себе помощь, а всем пациентам с гидроцеле была предложена бесплатная хирургия.

Выводы: Благодаря успеху МПЛ и развитию мощностей центров здоровья по оказанию помощи пациентам, наряду с плодами социально-экономических улучшений и других вмешательств, направленных против трансмиссивных болезней и ЗТБ, Всемирная организация здравоохранения признала, что Камбоджа в 2016 г. достигла устранения ЛФ в качестве проблемы общественного здравоохранения.

Translated from English version into Russian by Alexander Somin, through

Cómo se logró la eliminación de la filariasis linfática como un problema de salud pública en el Reino de Camboya

Virak Khieu, Vandine Or, Chhakda Tep , Peter Odermatt , Reiko Tsuyuoka , Meng Chuor Char , Molly A. Brady , Joshua Sidwell, Aya Yajima, Rekol Huy, Kapa D. Ramaiah y Sinuon Muth

Resumen

Antecedentes: el endemismo de la filariasis linfática (FL) en Camboya se demostró en 1956 cuando se detectaron microfilarias en mosquitos en la provincia de Kratié. En el 2001, un estudio extensivo confirmó la presencia tanto de microfiliarias *Brugia malayi* como de las *Wuchereria bancrofti.* En el 2003, con el objetivo de eliminar la LF para el 2015, el Ministrerio de Salud estableció una fuerza de tareas nacional para desarrollar políticas y estrategias para controlar y eliminar las enfermedades tropicales desatendidas (ETD). Este artículo resume el trabajo realizado para eliminar la FL como un problema de salud pública en Camboya.

Métodos: el Programa Nacional para Eliminar la Filariasis Linfática alcanzó un progreso excelente en el objetivo de lograr la eliminación, gracias a la estrecha colaboración entre los ministerios, la supervisión intensiva del personal nacional y el apoyo para movilizar recursos internos y externos. La administración masiva de medicamentos (AMM) con citrato de dietilcarbamazina y albendazol se llevó a cabo en seis unidades de implementación y alcanzó más de un 70% de cobertura epidemiológica en cinco rondas consecutivas, desde el 2005 hasta el 2009. En el 2006, en catorce provincias, trabajadores de la salud desarrollaron una lista de línea de pacientes con linfedema e hidrocele, muchos de los cuales eran mayores de 40 años y habían estado afectados por la FL durante muchos años. El programa nacional también entrenó a trabajadores de la salud y a personal provincial y distrital en la gestión de la morbidez y en la prevención de la discapacidad. Asimismo, entrenaron centros de salud designados para brindar atención para la linfedema y el ataque agudo. Se designaron dos hospitales para que realicen cirugías de hidrocele.

Resultados: se comprobó la efectividad de la AMM a través de encuestas de evaluación de la transmisión. Las mismas revelaron que menos del 1% de los escolares tenían antigenemia en el 2010, la que descendió a 0% tanto en el 2013 como en el 2015. Una encuesta por separado en una provincia en el 2015 que utilizó las prueba rápidas para Brugia para analizar si había presencia de anticuerpos contra la FL encontró un niño positivo entre 1 677 niños. La lista de pacientes crónicos de FL se actualizó recientemente y confirmó que en el período 2011–2012, se listaron treinta y dos pacientes con linfedema y diecisiete con hidrocele. Todos los pacientes con linfedema habían sido entrenados en la autogestión y a todos los pacientes con hidrocele se les ofreció cirugía gratuita.

Conclusiones: debido al éxito de la AMM y al desarrollo de la capacidad de centros de salud para la atención de pacientes, junto con los beneficios obtenidos por las mejoras socioeconómicas y otras intervenciones contra las enfermedades que portan el vector y las ETD, Camboya fue validada por la Organización Mundial para la Salud como que logró eliminar la FL como un problema de salud pública en el 2016.

Translated from English version into Spanish by Lidia Lariño, through
